# Aortoiliac diameter and length in a healthy cohort

**DOI:** 10.1371/journal.pone.0268077

**Published:** 2022-05-05

**Authors:** Hyangkyoung Kim, Tae-Won Kwon, Eol Choi, Seonjeong Jeong, Hong-Kyu Kim, Youngjin Han, Yong-Pil Cho, Hyun-Ki Yoon, Jaewon Choe, Won Hong Kim

**Affiliations:** 1 Department of Surgery, Kyung Hee University Hospital at Gangdong, Kyung Hee University School of Medicine, Seoul, Korea; 2 Division of Vascular Surgery, Department of Surgery, University of Ulsan, College of medicine and Asan Medical Center, Seoul, Korea; 3 Health Medicine, Health Screening & Promotion Center, University of Ulsan, College of medicine and Asan Medical Center, Seoul, Korea; 4 Department of Radiology, University of Ulsan, College of medicine and Asan Medical Center, Seoul, Korea; 5 Department of Radiology, College of Medicine, Inha University Hospital, Incheon, Korea; Leiden University Medical Center, NETHERLANDS

## Abstract

**Objective:**

Diameter is currently the only screening and diagnostic criterion for asymptomatic aneurysms. Therefore, aortic and lower-extremity arterial diameter has diagnostic, therapeutic, and prognostic importance. We aimed to determine aortic and lower-extremity arterial reference diameters in a general population and compare them according to age, sex, and other characteristics.

**Methods:**

We evaluated consecutive 3,692 patients who underwent computed tomography as part of a general health checkup from 2015–2019 in a single tertiary center. Aortic and lower-extremity arterial diameters and the most important factor related to arterial diameters were evaluated.

**Results:**

The mean diameter of the abdominal aorta was 17.490 ± 2.110 mm, while that of the common iliac artery was 10.851 ± 1.689 mm. The mean diameter of the abdominal aorta was 18.377 ± 1.766 mm in men and 15.884 ± 1.694 mm in women. Significant intersex differences were observed for all mean diameters and lengths. Multilinear regression analysis showed that age, sex, and body surface area impacted mean diameters of all measured sites except aorta and common iliac artery length. Between male and female patients matched for body surface area, there were significant intersex differences for all measured sites, except for common iliac artery length.

**Conclusions:**

The mean diameter of the abdominal aorta in this healthy cohort was 17.490 ± 2.110 mm overall, 18.377 ± 1.766 mm in men, and 15.884 ± 1.694 mm in women. Arterial diameter increased with male sex, older age, and increased body surface area, and aortic diameters were larger in men than in women with the same body surface area.

## Introduction

Currently, diameter is the only screening and diagnostic criterion for asymptomatic aneurysms. Abdominal aortic aneurysm (AAA) is defined as a 50% or greater increase in infrarenal aortic diameter (IAD) or infrarenal aorta with a maximum diameter ≥ 3.0 cm [1–3]. Aneurysm size is one of the strongest predictors for risk of rupture, with a markedly increased risk when aneurysm diameters are greater than 5.5 cm [4, 5]. Therefore, aortoiliac arterial diameter has diagnostic, therapeutic and prognostic importance.

Women have up to a four-fold higher risk of AAA rupture than men at any given aneurysm diameter [6]. The Joint Council of the American Association of Vascular Surgery and the Society for Vascular Surgery have suggested a lower diameter threshold for AAA repair in women [7]. One hypothesis is that because women generally have a smaller body and vascular size than men, an aneurysm of a certain size in a woman represents a greater relative dilatation of the aorta compared with the same aneurysm in a man [8]. In order to apply the concept of relative expansion according to sex or body size, the reference diameter is of clinical importance. There are published reference ranges for the aorta and the lower-extremity vessels using ultrasound or contrast-enhanced computed tomography (CT) [9–11]. However, there are few papers on Asian populations, the body sizes of whom are relatively small compared to Westerners. Considering its clinical importance and lack of sufficient data, we purposed to measure aortoiliac and lower-extremity arterial reference diameters in an Asian healthy population. In addition, we determined whether body size was a significant factor for aortoiliac diameter and whether there was an intersexual difference in the diameter when body size was similar.

## Methods

We retrospectively evaluated patients who underwent CT for general health checkups from 2015 to 2016. Patients with aneurysms or atherosclerotic plaques with or without calcification or patients whose arterial centerline could not be obtained were excluded from the analysis. This study was approved by _____ Institutional Review Board (No. 2016–0232) and waived the need for informed consent because of the retrospective nature of the study and the lack of information on the participant’s identification. This study complies with the Declaration of Helsinki.

All imaging examinations were performed using a multi-slice CT scanner (Lightspeed VCT; GE Healthcare, IL, US). Parameters for the acquisitions were 5-mm slice thickness, 120 KVp, and 215–360 mA tube current. Imaging was initiated after the administration of low osmolar iodinated contrast agent (Iopamiro 2 mL/kg; iodine concentration, 320 mg/mL). Soft-tissue window settings with a width of 300 HU and a center of 50 HU were applied. This sizing was performed using Endosize (Therenva, Rennes, France), a 3D sizing software tool that measures diameters perpendicular to the long axis of the arteries. Lengths and diameters taken on the vessel centerlines were automatically obtained after a simple interactive step consisting of a 3D point picking sequence.

**Fig 1:**
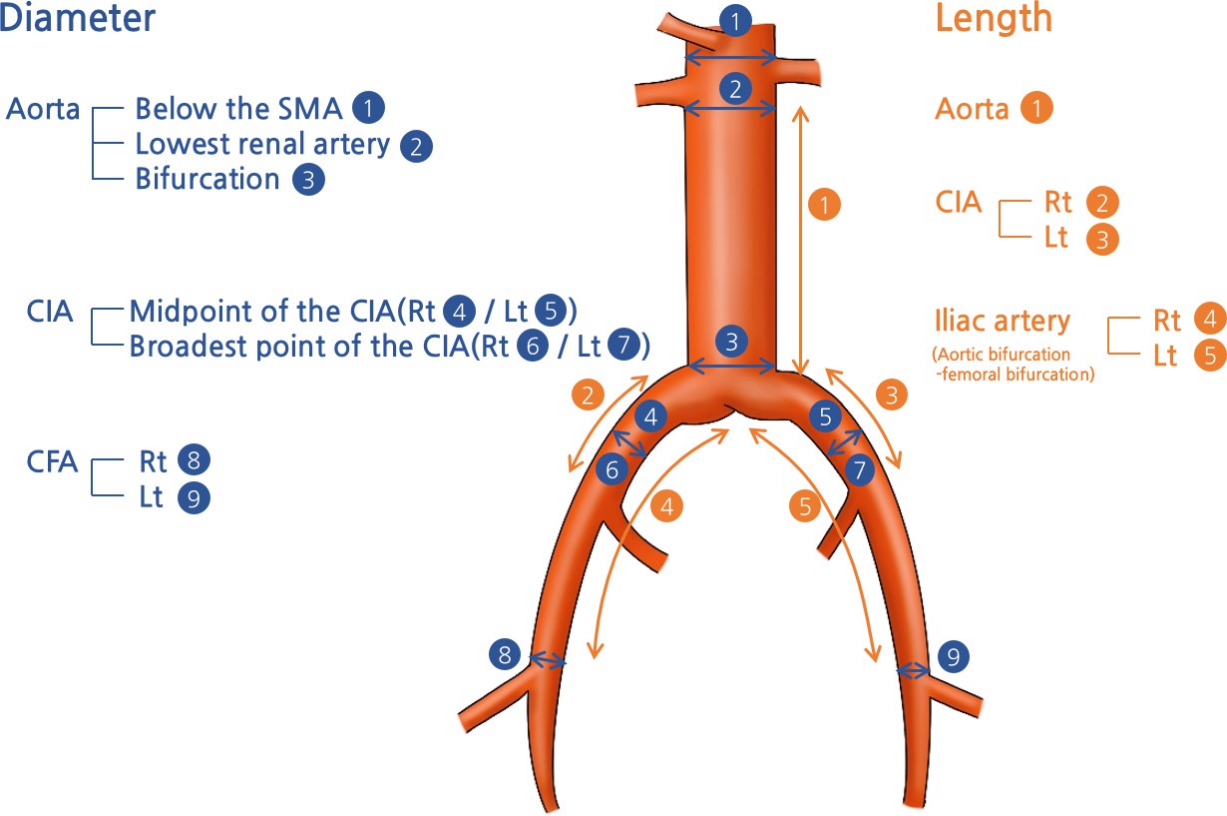
Diameter and length measurements at each site. SMA, superior mesenteric artery; CIA, common iliac artery; CFA, common femoral artery; Rt, right; Lt, left.

The measured site is depicted in Fig 1. Aortic diameter was measured just below the superior mesenteric artery (SMA), lowest renal artery, and at the bifurcation. Mean aortic diameter from three sites was used in the regression analysis. The diameter of the common iliac artery (CIA) was measured at the midpoint between the aortic and iliac bifurcation and at the broadest point, and the external iliac artery (EIA) diameter was measured at the iliac bifurcation. The diameter of the common femoral artery was measured at the level of the femoral bifurcation. The diameter of each artery was measured with the outer diameter of the artery perpendicular to the arterial centerline. Aortic length was measured between the lowest renal artery and the bifurcation. CIA length was measured between the aortic bifurcation and the iliac bifurcation. Iliac artery length was measured between the aortic bifurcation and the femoral bifurcation. Measurements using Endosize were made by four vascular surgeons. To test the reliability, all four of the examiners randomly measured the data of the selected 106 patients using a random number generation function in Microsoft Excel (Microsoft Corporation, Redmond, WA, USA).

Clinical information was obtained from the questionnaires and measurements from the general health checkup database, including height, weight, history of smoking, hypertension, and diabetes. History of smoking was defined as current or former smokers based on patient-provided information. Body mass index (BMI) was calculated by dividing the weight in kilograms by the square of the height in meters. Body surface area (BSA) was calculated using the Mosteller formula [12]. Diabetes was defined as fasting plasma glucose (FPG) levels ≥ 7.0 mmol/L or glycated hemoglobin (HbA1c) levels ≥ 6.5%. In addition, individuals taking anti-diabetic medication were considered to have diabetes. Hypertension was defined as systolic and/or diastolic blood pressure ≥ 140/90 mmHg and/or taking antihypertensive medication. The CT scan, laboratory tests, and questionnaire evaluations were performed on the same day or the following day.

### Statistical analysis

Quantitative and qualitative variables were summarized separately by descriptive statistics. For quantitative variables, an independent sample t-test or one-sample t-test was used to assess differences in the diameters. Inter-observer reliability of the measurements was assessed using the intraclass coefficient correlation (ICC), and complete agreement was defined as 1.0. A generalized linear model with stepwise selection was fit to assess the associations between baseline characteristics and the diameters of the lower extremities after normality testing (Kolmogorov-Smirnov test, Cramer-von Mises, and Anderson-Darling). Men and women with the same BSA were extracted using R software version 4.0.2 (R Development Core Team, 2006). Where multiple patients were present for one BSA value, the mean values of each sex were used as representative values. Comparison of aortic diameters was performed between matched men and women using paired t-tests. Our data were compared with those of previous studies on aortic diameters using one-sample t-test. p values < 0.05 were considered significant. The statistical analysis was performed using SAS software version 9.4 (SAS Institute Inc., Cary, NC, USA) and SPSS version 23.0 software (Armonk, NY, USA).

## Results

**Fig 2:**
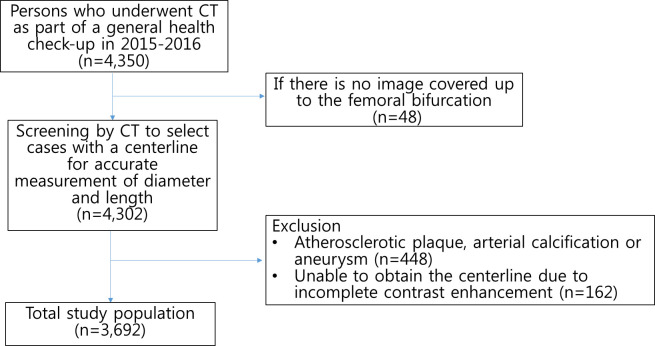
Flow diagram.

A total of 3,692 subjects (35.6% female) were included in the analysis (Fig 2). Baseline characteristics are summarized in Table 1. Mean age was 57.3 ± 8.7 years (range, 21–88 years) (median age, 57 years; 5% trimmed mean, 57.2 years). All ICC were above 0.9 except for aortic diameter at the bifurcation: aortic diameter at the SMA level, 97.3% [96.3%, 98.0%] (*P* < .001); aortic diameter at the lowest renal artery level, 93.9% [91.7%, 95.6%] (*P* < .001); aortic diameter at the bifurcation, 78.1% [69.7%, 84.4%] (*P* = .003); right CIA diameter, 91.1% [87.7%, 93.7%] (*P* < .001); left CIA diameter, 95.6% [93.9%, 96.9%] (*P* < .001); aortic length, 96.2% [94.9%, 96.3%] (*P* < .001); right CIA length, 97.8% [97.0, 98.4%] (*P* < .001); and left CIA length, 97.4% [96.4%, 98.1%] (*P* < .001).

**Table 1:** Patients’ demographic data.

	Total		Male	Female	P
Number	3,692		2,379 (64.4%)	1,313 (35.6%)	
Age	57.3 ± 8.7 (range, 21–88)		56.8 ± 8.7	58.1 ± 8.6	<0.001
Height	166.3 ± 8.4 (range, 137.4–191.7)		170.9 ± 5.8	157.8 ± 5.4	<0.001
BMI	24.33 ± 3.05 (range, 15.13–45.27)		25.0 ± 2.8	23.2 ± 3.06	<0.001
BSA	1.76 ± 0.19 (range, 1.19–2.77)		1.86 ± 0.15	1.59 ± 0.11	<0.001
Hypertension	1,106 (30%)		795 (33.4%)	344 (23.7%)	<0.001
DM	547 (14.8%)		434 (18.2%)	113 (8.6%)	<0.001
Smoking	Nonsmoker	1,689 (45.7%)	462 (19.4%)	1,227 (93.8%)	<0.001
	Current smoker	749 (20.3%)	713 (30.0%)	36 (2.8%)	
	Ex-smoker	1,247 (33.8%)	1,202 (50.6%)	45 (3.4%)	
CVD	1,633 (44.2%)		1,187 (49.9%)	446 (34.0%)	<0.001
BUN	13.12 ± 3.54 (range, 3–30)		13.5 ± 3.4	12.4 ± 3.6	<0.001
Creatinine	0.85 ± 0.17 (range, 0.4–1.44)		0.93 ± 0.1	0.69 ± 0.1	<0.001
eGFR	90.81 ± 11.96 (range, 50–127)		89.5 ± 12.0	93.2 ± 11.5	<0.001
HbA1c	5.78 ± 0.81 (range, 4.0–13.2)		5.8 ± 0.9	5.7 ± 0.7	<0.001
Cholesterol	185.62 ± 40.15 (range, 78–385)		181.6 ± 40.4	192.5 ± 38.7	<0.001
Triglyceride	123.54 ± 84.93 (range, 13–1190)		136.8 ± 94.1	99.5 ± 57.9	<0.001
HDL	55.75 ± 16.02 (range, 19–185)		51.9 ± 14.0	62.7 ± 17.0	<0.001
LDL	127.29 ± 37.39 (range, 32–316)		125.5 ± 37.9	130.5 ± 36.2	<0.001

BMI, body mass index; BSA, body surface area; BUN, blood urea nitrogen; CVD, any type of cardiovascular disease; DM, diabetes mellitus; eGFR, estimated glomerular filtration rate; HDL, high-density lipoprotein cholesterol; LDL, low-density lipoprotein cholesterol

### Mean diameters and lengths

**Fig 3:**
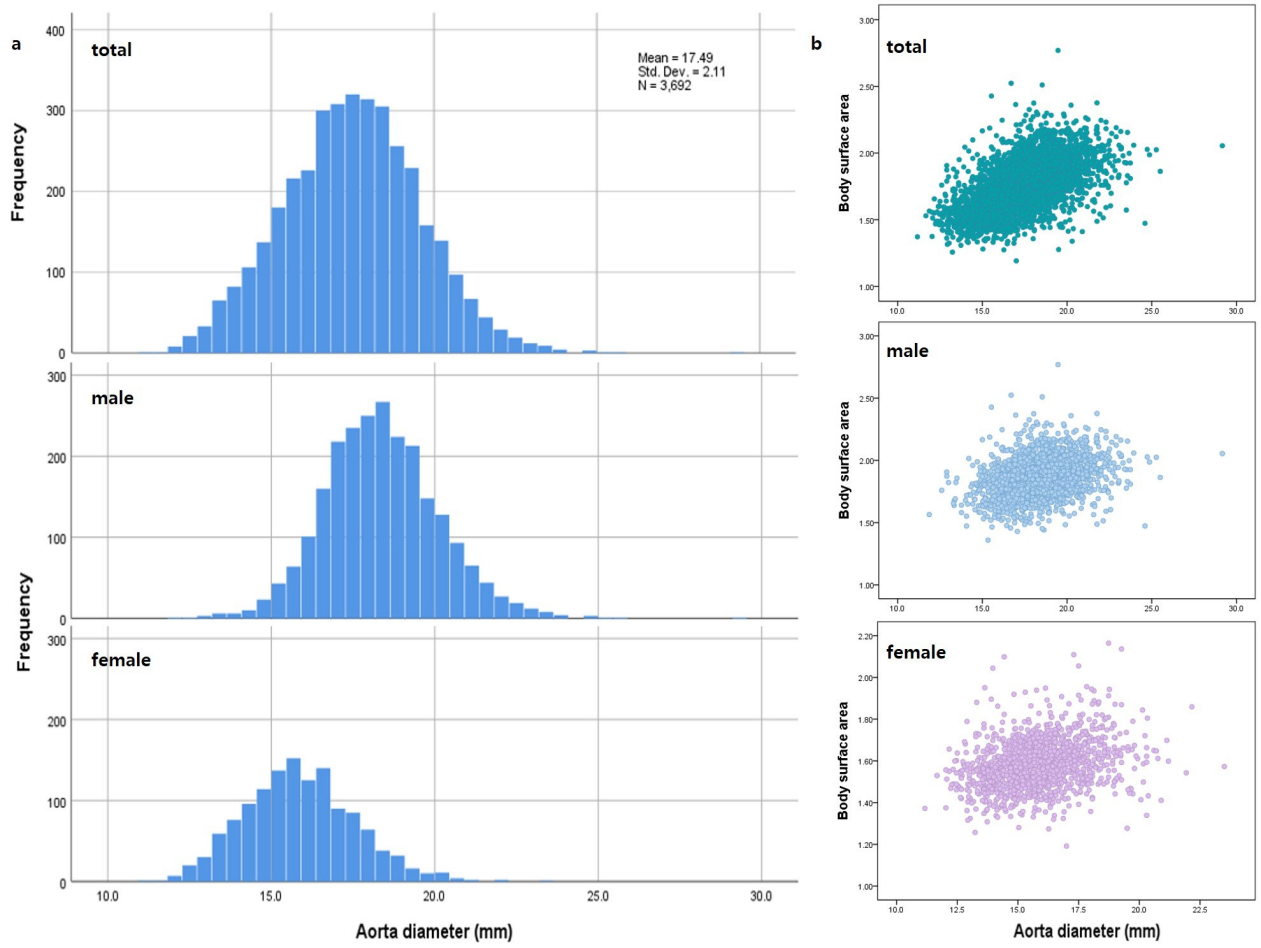
Distribution of aortic diameters by sex (A) and body surface area (B).

Mean diameters and lengths are shown in Table 2. The mean diameter of the abdominal aorta was 17.490 ± 2.110 mm, while that of the CIA was 10.851 ± 1.689 mm. No patients had an aortic diameter ≥ 3 cm. The mean diameter of the aorta was 18.377 ± 1.766 mm in men and 15.884 ± 1.694 mm in women (Fig 3). The mean diameter of the CIA was 11.436 ± 1.512 mm in men and 9.793 ± 1.464 mm in women. For all mean diameters and lengths, significant differences between men and women were observed (all p values were less than 0.001, except for both CIA lengths [*P* = .048 for right side, *P* = .034 for left side]).

**Table 2:** Arterial diameters and lengths.

Location	Overall	Interquartile range	Men	Women
			Mean (mm) ± SD	Mean (mm) ± SD
	Mean (mm) ± SD			
Aorta, SMA level	19.116 ± 2.490	17.4–20.7	20.080 ± 2.125	17.368 ± 2.123
Aorta, lowest. renal (D)	17.014 ± 2.301	15.5–18.6	17.867 ± 2.041	15.469 ± 1.909
Aorta, bifurcation (D)	16.340 ± 2.240	14.8–17.6	17.183 ± 2.012	14.814 ± 1.783
Mean aorta (D)	17.490 ± 2.110	16.07–18.90	18.377 ± 1.766	15.884 ± 1.694
Mean aorta (L)	92.977 ± 13.436	30.0–145.0	94.271 ± 13.620	90.620 ± 12.750
Rt. CIA, max (D)	11.376 ± 2.028	10.0–12.6	11.970 ± 1.898	10.303 ± 1.800
Lt. CIA, max (D)	11.175 ± 1.994	9.8–12.4	11.784 ± 1.855	10.073 ± 1.751
Rt. CIA, mid (D)	10.494 ± 1.851	9.2–12.4	11.065 ± 1.714	9.460 ± 1.628
Lt. CIA, mid (D)	10.359 ± 1.812	9.1–11.5	10.924 ± 1.684	9.334 ± 1.572
Mean CIA (D)	10.851 ± 1.689	9.7–11.9	11.436 ± 1.512	9.793 ± 1.464
Rt. CIA (L)	48.656 ± 15.170	9.0–106.0	49.029 ± 15.174	47.994 ± 15.151
Lt. CIA (L)	53.493 ± 16.257	4.0–121.0	53.926 ± 16.286	52.726 ± 16.173
Mean CIA (L)	51.075 ± 14.407	41.5–60.0	51.478 ± 14.470	50.360 ± 14.269
Rt. CFA (D)	9.015 ± 1.379	8.1–9.9	9.529 ± 1.220	8.085 ± 1.143
Lt. CFA (D)	9.018 ± 1.426	8.1–10.0	9.529 ± 1.220	8.085 ± 1.143
Mean CFA (D)	9.017 ± 1.340	8.1–9.9	9.529 ± 1.170	8.091 ± 1.111
Bifurcation, Rt. SFA (L)	211.455 ± 19.427	120.0–306.0	215.805 ± 19.082	203.598 ± 17.462
Bifurcation, Lt. SFA (L)	207.891 ± 19.483	109.0–283.0	212.575 ± 18.778	199.431 ± 17.789
Mean Iliac artery (L)	209.673 ± 18.319	196.5–221.5	10.800 ± 1.307	9.225 ± 1.269
Bifurcation Rt. SFA in COR (L)	188.007 ± 17.085	176.7–198.5	190.505 ± 17.068	183.500 ± 16.164
Bifurcation Lt. SFA in COR (L)	185.193 ± 17.300	173.8–197.1	188.123 ± 16.750	179.881 ± 17.013

CFA, common femoral artery; CIA, common iliac artery; COR, coronal plane; (D), diameter; IQR, interquartile range; (L), length; Lt., left; Rt., right; SFA, superior mesenteric artery; SMA, superior mesenteric artery

### Factors affecting diameters and lengths

The linear and multilinear regression analyses for each variable were performed for each diameter and length (Tables 3 and 4). The results of multilinear regression showed that age, sex, and BSA were related to the mean diameters and lengths of all measured sites except for CIA length. In the linear regression model, BSA was most explanatory for diameters with the highest *R*^2^ values; 0.249 for the infrarenal aorta, 0.277 for the lower abdominal aorta near the bifurcation, 0.217 for the CIA, and 0.254 for the iliac artery. The female sex variable further exhibited high *R*^2^ values; 0.249 for the infrarenal aorta, 0.256 for the lower abdominal aorta near the bifurcation, 0.257 for the CIA, and 0.296 for the iliac artery. Results of the multilinear regression analysis with significant variables in the linear regression analysis showed that age, sex, and BSA were related to mean diameters of all measured sites. The *R*^2^ value of the reduced model regarding only age, sex, and BSA was not significantly different from that of the full model including all possible variables that were significant in the linear regression; F_(3,3687)_ = 735.859, *P* < .001, *R*^2^ = 0.375 vs F_(6,3684)_ = 372.696, *P* < .001, *R*^2^ = 0.378 for the infrarenal aorta, F_(3,3687)_ = 694.286, *P* < .001, *R*^2^ = 0.361 vs F_(6,3684)_ = 354.514, *P* < .001, *R*^2^ = 0.366 for the lower abdominal aorta, F_(3,3687)_ = 640.117, *P* < .001, *R*^2^ = 0.345 vs F_(6,3684)_ = 326.196, *P* < .001, *R*^2^ = 0.347 for the CIA, F_(3,3687)_ = 735.448, *P* < .001, *R*^2^ = 0.374 vs F_(5,3685)_ = 451.133, *P* < .001, *R*^2^ = 0.380 for the iliac artery.

**Table 3:** Linear and multilinear regression of aortic diameters and lengths with variables.

		Linear Regression			Multilinear Regression	
	Variable	Coeff.	R^2^	P	Coeff.	P
Infrarenal aorta diameter	Female sex	-2.399	0.249	< .0001	-1.008	< .0001
	Age	0.048	0.033	< .0001	0.080	< .0001
	Height*	0.120	0.194	< .0001		
	Weight*	0.093	0.229	< .0001		
	Body mass index*	0.246	0.105	< .0001		
	Body surface area	6.077	0.249	< .0001	5.050	< .0001
	Smoking (ref. = non-smoker)					
	Current smoker	0.854	0.022	< .0001	0.340	0.002
	Ex-smoker	1.295	0.071	< .0001		0.237
	Hypertension	0.770	0.024	< .0001		0.155
	DM	0.484	0.006	< .0001	-0.173	0.044
	HbA1c	0.237	0.007	< .0001		0.665
	Cholesterol	-0.007	0.015	< .0001		0.667
	Triglyceride	0.002	0.008	< .0001	0.001	0.003
Lower abdominal aorta diameter	Female sex	-2.369	0.256	< .0001	-1.062	< .0001
	Age	0.027	0.011	< .0001	0.056	< .0001
	Height*	0.123	0.215	< .0001		
	Weight*	0.096	0.257	< .0001		
	Body mass index*	0.254	0.117	< .0001		
	Body surface area	6.249	0.277	< .0001	5.137	< .0001
	Current smoker	0.798	0.021	< .0001		0.146
	Ex-smoker	1.296	0.075	< .0001		0.909
	Hypertension	0.721	0.022	< .0001		0.127
	DM	0.457	0.005	< .0001		0.883
	HbA1c	0.138	0.002	0.002	-0.132	< .0001
	Cholesterol	-0.008	0.020	< .0001	-0.002	0.003
	Triglyceride	0.002	0.009	< .0001	-0.001	0.030
Aortic Length	Female sex	-3.670	0.017	< .0001		0.798
	Age	0.157	0.010	< .0001	0.240	< .0001
	Height*	0.309	0.038	< .0001		
	Weight*	0.191	0.028	< .0001		
	Body surface area	13.123	0.034	< .0001	15.781	< .0001
	Smoking (ref. = non-smoker)					
	Current smoker			0.448		
	Ex-smoker	2.611	0.008	< .0001		0.753
	Hypertension	1.498	0.003	0.002		0.463
	DM	1.387	0.001	0.026		0.928
	HbA1c	0.668	0.002	0.014		0.833
	Cholesterol	-0.021	0.004	< .0001		0.283
	Triglyceride	0.005	0.001	0.045		0.667

**Table 4:** Linear and multilinear regression of Common Iliac Artery (CIA) and iliac artery (common iliac to external iliac artery) diameters and lengths with variables.

		Linear Regression			Multilinear Regression	
	Variable	Coeff.	R^2^	P	Coeff.	P
CIA Diameter	Female sex	-1.644	0.217	< .0001	-0.621	< .0001
	Age	0.026	0.018	< .0001	0.048	< .0001
	Height*	0.084	0.175	< .0001		
	Weight*	0.071	0.246	< .0001		
	Body surface area	4.533	0.257	< .0001	4.099	< .0001
	Smoking (ref. = non-smoker)					
	Current smoker	0.452	0.012	< .0001		0.932
	Ex-smoker	0.967	0.073	< .0001		0.332
	Hypertension	0.581	0.025	< .0001	0.122	0.018
	DM	0.344	0.005	< .0001		0.954
	HbA1c	0.131	0.004	< .0001	-0.067	0.020
	Cholesterol	-0.005	0.015	< .0001		0.557
	Triglyceride	0.002	0.006	< .0001	-0.001	< .0001
CIA Length	Female sex	-1.107	0.001	0.025		0.369
	Age	-0.023	0.000	0.410		
	Height*	0.124	0.005	< .0001		
	Weight*	0.078	0.004	< .0001		
	Body surface area	5.222	0.005	< .0001	5.222	< .0001
	Smoking (ref. = non-smoker)					
	Current smoker			0.372		
	Ex-smoker	0.876	0.001	0.081		0.710
	Hypertension			0.881		
	DM			0.992		
	HbA1c			0.267		
	Cholesterol			0.193		
	Triglyceride			0.898		
Iliac artery Diameter	Female sex	-1.575	0.254	< .0001	-0.636	< .0001
	Age	0.018	0.010	< .0001	0.040	< .0001
	Height*	0.083	0.217	< .0001		
	Weight*	0.067	0.278	< .0001		
	Body surface area	4.314	0.296	< .0001	3.794	< .0001
	Smoking (ref. = non-smoker)					
	Current smoker	0.411	0.012	< .0001		
	Ex-smoker	0.930	0.086	< .0001		
	Hypertension	0.474	0.021	< .0001		
	DM	0.237	0.003	0.001		

	HbA1c	0.077	0.002	0.011	-0.093	< .0001
	Cholesterol	-0.004	0.014	< .0001		
	Triglyceride	0.002	0.008	< .0001	-0.001	< .0001
Iliac artery Length	Female sex	-12.685	0.110	< .0001	-3.281	< .0001
	Age	0.098	0.002	< .0001	0.275	< .0001
	Height*	0.905	0.173	< .0001		
	Weight*	0.583	0.142	< .0001		
	Body surface area	39.489	0.166	< .0001	39.661	< .0001
	Smoking (ref. = non-smoker)					
	Current smoker	1.840	0.002	0.014	-2.431	0.001
	Ex-smoker	7.780	0.040	< .0001		0.529
	Hypertension	4.211	0.011	< .0001		0.280
	DM	1.591	0.001	0.062		0.125
	HbA1c			0.921		
	Cholesterol	-0.037	0.007	< .0001		0.782
	Triglyceride	0.010	0.002	0.003	-0.011	0.001

Coeff., regression coefficient; ref, reference; DM, diabetes mellitus; HbA1c, glycated hemoglobin

*Height, weight, and body mass index were not used for the multilinear analysis due to multicollinearity.

### Difference between men and women in diameter and length when matching BSA

**Fig 4:**
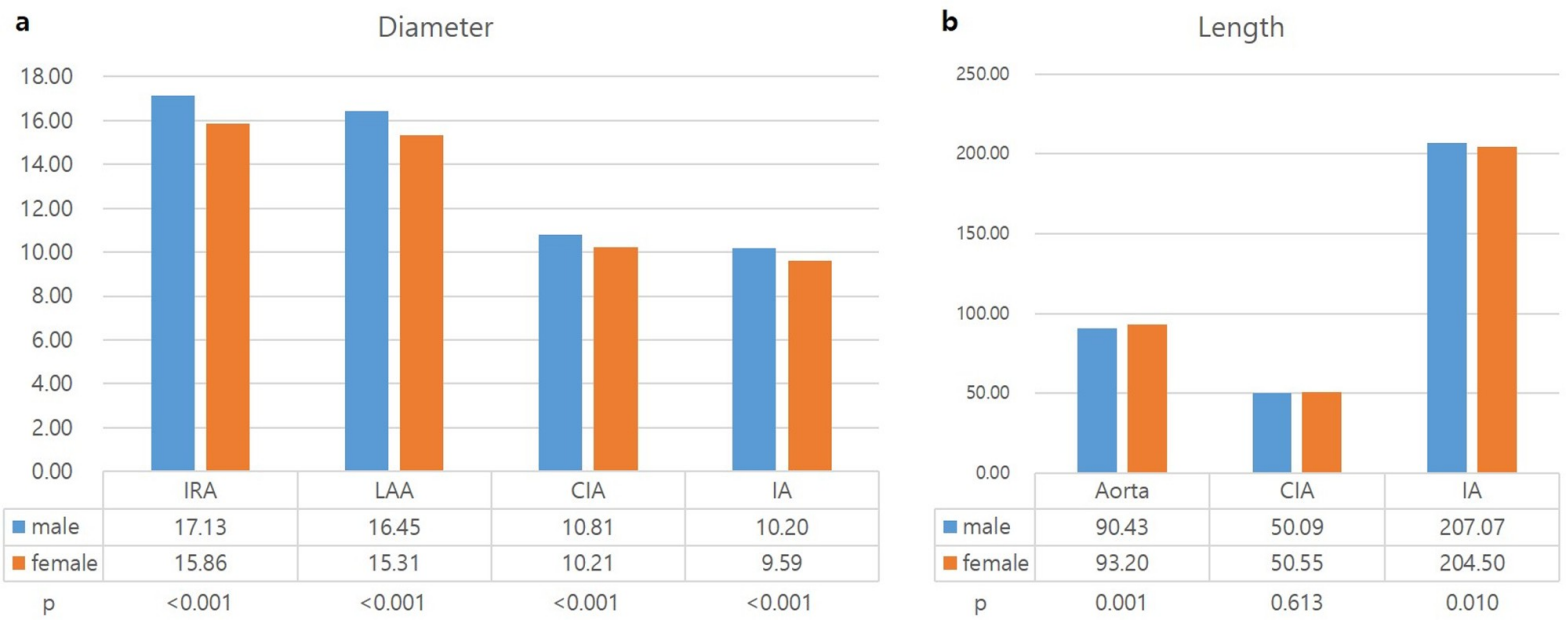
Comparison of aortic diameters between men and women with the same body surface area. IRA, infrarenal aorta; LAA, lower abdominal aorta; CIA, common iliac artery; IA, iliac artery.

When BSAs of men and women were matched, a total of 462 pairs were obtained (BSA range, 1.4–2.2). There was a significant difference in diameters between matched men and women (*P* < .05, Fig 4A). The difference between men and women was 1.26 [95% CI, 1.03–1.50] for the infrarenal aorta, 1.14 [95% CI, 0.60–1.38] for the lower abdominal aorta, 0.60 [95% CI, 0.42–0.78] for the CIA, and 0.62 [95% CI, 0.46–0.77] for the iliac artery. The length of the aorta was significantly longer in women (*P* = .001), while the length of the iliac artery was longer in men (*P* = .010) (Fig 4B). The difference in the aortic length and iliac artery was 2.77 [95% CI, 1.13–4.4] and 2.58 [95% CI, 0.62–4.53], respectively. There was no significant difference in CIA length (*P* = .613, Fig 4B).

## Discussion

Aorta and iliac artery size are considered essential in the diagnosis of aneurysm and the prediction of future aneurysmal rupture. The generally accepted definition of arterial aneurysm is a focal and persistent vessel dilation of 150% or more versus the expected normal diameter of the artery in question [1]. An association between age, sex, and body surface area and the normal diameter of the artery was proposed, but simpler definitions were then suggested since the effect on aortic diameter was not substantial [13]. Previous studies demonstrated that the normal IAD is slightly less than 20 mm in elderly men [1, 14]. Accordingly, AAA in this population was defined as an IAD ≥ 30 mm [15].

AAA is usually asymptomatic until rupture, and mortality can reach 85–90% in cases of rupture [16]. Several large studies have shown that screening for this condition reduces aneurysm-related mortality [17, 18], and it is recommended in European guidelines for all elderly men and in American guidelines for elderly women and men with a history of tobacco use [19, 20]. The frequency of follow-up imaging depends on initial artery diameter, considering the increased risk of rupture [20]. In addition to a large initial aneurysm diameter, female sex is a known independent risk factor associated with rupture as well as a worse outcome [21–23]. Interestingly, rupture occurs at aneurysm diameters of 5 to 10 mm smaller in women than in men [24]. One of the potential reasons is that an aneurysm of a given diameter in women with relatively smaller aortas due to smaller body size represents a greater relative dilatation and thus more advanced disease of the aorta than an aneurysm of the same diameter in men [25]. Therefore, it seems crucial that we identify the reference value of the IAD, particularly according to sex.

In our study on healthy Asian cohorts, mean aortic diameter was 17.490 ± 2.110 mm. When divided by sex, mean diameter of the aorta was 18.377 ± 1.766 mm in men and 15.884 ± 1.694 mm in women. The difference in mean value between them was 2.493 mm, larger than the previous report of 1.4 mm from the Veterans Affairs Cooperative Study [26]. As BSA was significantly larger in men (*P* < .001) and was the strongest factor that affected vessel diameter in our regression model (*P* < 0.001), we matched BSA to determine whether a difference in the diameter between sex was derived from BSA difference. Even after BSA was corrected, the difference in the diameter between men and women remained in all measured diameters (*P* < 0.001). Therefore, considering intersex differences in the diagnosis of diseases related to arterial diameter seems necessary.

The comparison of our data with those of previous reports from other countries using a one-sample *t* test revealed significant differences. The Veterans Affairs Cooperative Study reported that the aortic diameters measured below and above the renal arteries on ultrasonography for male patients were 20 ± 3 and 21 ± 3 mm, respectively [26]. When we compared those values with our data on diameters measured at the levels of the SMA and lowest renal artery, our data were significantly smaller than both diameters (p < 0.001 for both). The mean infrarenal abdominal aortic diameters on CT scan in the Framingham Heart Study for men and women were 19.3 ± 2.9 and 16.7 ± 1.8 mm, respectively, which were significantly larger than our values (p < 0.001 for both) [27]. The mean aortic diameter at the bifurcation level was 18.7 ± 2.7 mm for men and 16 ± 1.7 mm for women, significantly larger than our values (p < 0.001 for both) [27]. In a study of a Turkish population, on ultrasonography, the mean subdiaphragmatic aortic diameters were 18 ± 3 mm for women and 19 ± 4 mm for men, while the mean aortic diameters at the bifurcation level were 15 ± 3 mm for women and 16 ± 4 mm for men [28]. Compared with the diameter at the level of the SMA and bifurcation, the mean diameter in women was significantly smaller than that in men in our study (p < 0.001 for all). In an Indian study, the mean diameters of the suprarenal and infrarenal abdominal aortas measured at the T12 and L3 vertebral levels on CT scan were 19.0 ± 2.3 and 13.8 ± 1.9 mm for men and 17.1 ± 2.3 and 12.0 ± 1.6 mm for women, respectively [29]. Compared with the diameter at the level of the SMA and bifurcation, all the values were significantly larger in our study (p < 0.001 for all). In a Chinese population, the inner diameter of the infrarenal aorta on CT scan was 16.49 ± 2.12 mm for men and 14.50 ± 1.73 mm for women; all the values in our study were significantly larger than these results (p < 0.001 for all) [30]. These results demonstrate differences among geographic regions. However, this finding is limited because the comparisons did not involve equal modalities and included anatomical levels with different measurements. An aneurysm diameter measured on standard axial CT is generally > 2 mm larger than when measured on ultrasonography [20]. Moreover, the actual difference was ≤ 2.5 mm. For example, the difference between the data from our study and those from the Framingham Heart Study was < 1 mm (0.92 mm for men and 0.81 mm for women) despite the statistical significance [27]. The clinical significance requires reevaluation with regard to the actual risk of rupture and the establishment of different surveillance criteria.

This study has some limitations. A potential source of bias in our study was selection bias because the study population consisted of people who prioritize their health status maintenance and included CT in their health checkups. However, we acknowledge that the selection bias in this study would be much lower than that if the data of CT scans conducted for patients with certain diseases were included. Moreover, we tried to overcome the selection bias by using a large sample size. Second, an unequal number of men and women were included since consecutive persons were enrolled. Despite these limitations, the strength of our study was that we used data from a healthy population without atherosclerotic steno-occlusive disease on CT scans. Because the artery tends to gets larger with the progression of the atherosclerotic disease; thus, the reference diameter needs to be evaluated from the normal population. Under the Korean health insurance system, people can opt to undergo a CT scan as part of their medical checkup. This is why we could obtain data from normal subjects for this analysis. Second, we investigated intersex difference in diameters with excluding the effect of BSA based on the large sample size. Lastly, we used 3D reconstruction to extract a centerline, avoid a parallax error, and increase reproducibility. When we evaluated intraobserver variability, reproducibility proved relatively efficient for obtaining reliable sizing data.

In conclusion, we obtained the reference diameters of the abdominal aorta of 17.490 ± 2.110 mm overall, 18.377 ± 1.766 mm in men, and 15.884 ± 1.694 mm in women in a Korean healthy cohort, which was smaller than Westerners. Arterial diameter increased with male sex, older age, and increased BSA, and the aortic diameters were larger in men than in women with the same BSA.

## Supporting information

S1 Data (XLSX)

## References

[1] Johnston KW, Rutherford RB, Tilson MD, Shah DM, Hollier L, Stanley JC. Suggested standards for reporting on arterial aneurysms. Subcommittee on Reporting Standards for Arterial Aneurysms, Ad Hoc Committee on Reporting Standards, Society for Vascular Surgery and North American Chapter, International Society for Cardiovascular Surgery. J Vasc Surg. 1991;13(3):452–8. doi: 10.1067/mva.1991.26737 1999868

[2] Hirsch AT, Haskal ZJ, Hertzer NR, Bakal CW, Creager MA, Halperin JL, et al. ACC/AHA 2005 Practice Guidelines for the management of patients with peripheral arterial disease (lower extremity, renal, mesenteric, and abdominal aortic): a collaborative report from the American Association for Vascular Surgery/Society for Vascular Surgery, Society for Cardiovascular Angiography and Interventions, Society for Vascular Medicine and Biology, Society of Interventional Radiology, and the ACC/AHA Task Force on Practice Guidelines (Writing Committee to Develop Guidelines for the Management of Patients With Peripheral Arterial Disease): endorsed by the American Association of Cardiovascular and Pulmonary Rehabilitation; National Heart, Lung, and Blood Institute; Society for Vascular Nursing; TransAtlantic Inter-Society Consensus; and Vascular Disease Foundation. Circulation. 2006;113(11):e463–654. doi: 10.1161/CIRCULATIONAHA.106.174526 16549646

[3] Chaikof EL, Brewster DC, Dalman RL, Makaroun MS, Illig KA, Sicard GA, et al. The care of patients with an abdominal aortic aneurysm: the Society for Vascular Surgery practice guidelines. J Vasc Surg. 2009;50(4 Suppl):S2–49. doi: 10.1016/j.jvs.2009.07.002 19786250

[4] Mortality results for randomised controlled trial of early elective surgery or ultrasonographic surveillance for small abdominal aortic aneurysms. The UK Small Aneurysm Trial Participants. Lancet. 1998;352(9141):1649–55. 9853436

[5] Lederle FA, Johnson GR, Wilson SE, Ballard DJ, Jordan WD Jr., Blebea J, et al. Rupture rate of large abdominal aortic aneurysms in patients refusing or unfit for elective repair. Jama. 2002;287(22):2968–72. doi: 10.1001/jama.287.22.2968 12052126

[6] Powell JT, Brady AR, Brown LC, Fowkes FG, Greenhalgh RM, Ruckley CV, et al. Long-term outcomes of immediate repair compared with surveillance of small abdominal aortic aneurysms. The New England journal of medicine. 2002;346(19):1445–52. doi: 10.1056/NEJMoa013527 12000814

[7] Brewster DC, Cronenwett JL, Hallett JW, Johnston KW, Krupski WC, Matsumura JS. Guidelines for the treatment of abdominal aortic aneurysms—Report of a subcommittee of the Joint Council of the American Association for Vascular Surgery and Society for Vascular Surgery. Journal of Vascular Surgery. 2003;37(5):1106–17. doi: 10.1067/mva.2003.363 12756363

[8] Lo RC, Lu B, Fokkema MT, Conrad M, Patel VI, Fillinger M, et al. Relative importance of aneurysm diameter and body size for predicting abdominal aortic aneurysm rupture in men and women. J Vasc Surg. 2014;59(5):1209–16. doi: 10.1016/j.jvs.2013.10.104 24388278 PMC4004688

[9] Joh JH, Ahn H-J, Park H-C. Reference diameters of the abdominal aorta and iliac arteries in the Korean population. Yonsei Med J. 2013;54(1):48–54. doi: 10.3349/ymj.2013.54.1.48 23225798 PMC3521285

[10] Lorbeer R, Grotz A, Dorr M, Volzke H, Lieb W, Kuhn JP, et al. Reference values of vessel diameters, stenosis prevalence, and arterial variations of the lower limb arteries in a male population sample using contrast-enhanced MR angiography. PloS one. 2018;13(6):e0197559. doi: 10.1371/journal.pone.0197559 29924802 PMC6010244

[11] Pedersen OM, Aslaksen A, Vik-Mo H. Ultrasound measurement of the luminal diameter of the abdominal aorta and iliac arteries in patients without vascular disease. Journal of Vascular Surgery. 1993;17(3):596–601. doi: 10.1067/mva.1993.39525 8445758

[12] Mosteller RD. Simplified calculation of body-surface area. The New England journal of medicine. 1987;317(17):1098. doi: 10.1056/NEJM198710223171717 3657876

[13] Lederle FA, Johnson GR, Wilson SE, Chute EP, Littooy FN, Bandyk D, et al. Prevalence and associations of abdominal aortic aneurysm detected through screening. Aneurysm Detection and Management (ADAM) Veterans Affairs Cooperative Study Group. Annals of internal medicine. 1997;126(6):441–9. doi: 10.7326/0003-4819-126-6-199703150-00004 9072929

[14] Evans GH, Stansby G, Hamilton G. Suggested standards for reporting on arterial aneurysms. J Vasc Surg. 1992;15(2):456. doi: 10.1016/0741-5214(92)90269-e 1590838

[15] Starck J, Aaltonen HL, Bjorses K, Lundgren F, Gottsater A, Sonesson B, et al. A significant correlation between body surface area and infrarenal aortic diameter is detected in a large screening population with possibly clinical implications. International angiology: a journal of the International Union of Angiology. 2019;38(5):395–401. 31560186 10.23736/S0392-9590.19.04071-9

[16] Kent KC. Clinical practice. Abdominal aortic aneurysms. The New England journal of medicine. 2014;371(22):2101–8. doi: 10.1056/NEJMcp1401430 25427112

[17] Legemate DA. Population screening reduces mortality rate from aortic aneurysm in men. The British journal of surgery. 2000;87(12):1734. doi: 10.1046/j.1365-2168.2000.01689-6.x 11123164

[18] Lindholt JS, Juul S, Fasting H, Henneberg EW. Hospital costs and benefits of screening for abdominal aortic aneurysms. Results from a randomised population screening trial. European journal of vascular and endovascular surgery: the official journal of the European Society for Vascular Surgery. 2002;23(1):55–60. doi: 10.1053/ejvs.2001.1534 11748949

[19] Wanhainen A, Verzini F, Van Herzeele I, Allaire E, Bown M, Cohnert T, et al. Editor’s Choice—European Society for Vascular Surgery (ESVS) 2019 Clinical Practice Guidelines on the Management of Abdominal Aorto-iliac Artery Aneurysms. European journal of vascular and endovascular surgery: the official journal of the European Society for Vascular Surgery. 2019;57(1):8–93. doi: 10.1016/j.ejvs.2018.09.020 30528142

[20] Chaikof EL, Dalman RL, Eskandari MK, Jackson BM, Lee WA, Mansour MA, et al. The Society for Vascular Surgery practice guidelines on the care of patients with an abdominal aortic aneurysm. J Vasc Surg. 2018;67(1):2–77 e2. 10.1016/j.jvs.2017.10.044 29268916

[21] Brown PM, Zelt DT, Sobolev B. The risk of rupture in untreated aneurysms: The impact of size, gender, and expansion rate. Journal of Vascular Surgery. 2003;37(2):280–4. doi: 10.1067/mva.2003.119 12563196

[22] Norman PE, Powell JT. Abdominal aortic aneurysm: the prognosis in women is worse than in men. Circulation. 2007;115(22):2865–9. doi: 10.1161/CIRCULATIONAHA.106.671859 17548742

[23] Brown LC, Powell JT, with TUKSATP. Risk Factors for Aneurysm Rupture in Patients Kept Under Ultrasound Surveillance. Annals of Surgery. 1999;230(3):289. doi: 10.1097/00000658-199909000-00002 10493476 PMC1420874

[24] Lo RC, Lu B, Fokkema MTM, Conrad M, Patel VI, Fillinger M, et al. Relative importance of aneurysm diameter and body size for predicting abdominal aortic aneurysm rupture in men and women. Journal of Vascular Surgery. 2014;59(5):1209–16. doi: 10.1016/j.jvs.2013.10.104 24388278 PMC4004688

[25] Forbes TL, Lawlor DK, DeRose G, Harris KA. Gender Differences in Relative Dilatation of Abdominal Aortic Aneurysms. Annals of vascular surgery. 2006;20(5):564–8. doi: 10.1007/s10016-006-9079-y 16741651

[26] Lederle FA, Johnson GR, Wilson SE, Gordon IL, Chute EP, Littooy FN, et al. Relationship of age, gender, race, and body size to infrarenal aortic diameter. The Aneurysm Detection and Management (ADAM) Veterans Affairs Cooperative Study Investigators. J Vasc Surg. 1997;26(4):595–601. doi: 10.1016/s0741-5214(97)70057-0 9357459

[27] Rogers IS, Massaro JM, Truong QA, Mahabadi AA, Kriegel MF, Fox CS, et al. Distribution, determinants, and normal reference values of thoracic and abdominal aortic diameters by computed tomography (from the Framingham Heart Study). Am J Cardiol. 2013;111(10):1510–6. doi: 10.1016/j.amjcard.2013.01.306 23497775 PMC3644324

[28] Sariosmanoglu N, Ugurlu B, Karacelik M, Tuzun E, Yorulmaz I, Manisali M, et al. A multicentre study of abdominal aorta diameters in a Turkish population. J Int Med Res. 2002;30(1):1–8. doi: 10.1177/147323000203000101 11921493

[29] Jasper A, Harshe G, Keshava SN, Kulkarni G, Stephen E, Agarwal S. Evaluation of normal abdominal aortic diameters in the Indian population using computed tomography. Journal of postgraduate medicine. 2014;60(1):57–60. doi: 10.4103/0022-3859.128813 24625941

[30] Wang X, Zhao WJ, Shen Y, Zhang RL. Normal Diameter and Growth Rate of Infrarenal Aorta and Common Iliac Artery in Chinese Population Measured by Contrast-Enhanced Computed Tomography. Annals of vascular surgery. 2019. doi: 10.1016/j.avsg.2019.05.030 31394221

